# Long-term efficacy of BCG vaccination in goat herds with a high prevalence of tuberculosis

**DOI:** 10.1038/s41598-020-77334-1

**Published:** 2020-11-23

**Authors:** Claudia Arrieta-Villegas, Alberto Allepuz, Miriam Grasa, Maite Martín, Zoraida Cervera, Irene Mercader, Sergio López-Soria, Mariano Domingo, Bernat Pérez de Val

**Affiliations:** 1 IRTA, Centre de Recerca en Sanitat Animal (CReSA, IRTA-UAB), Campus UAB, Bellaterra, Barcelona, Catalonia Spain; 2 Departament de Sanitat i Anatomia Animals, Universitat Autònoma de Barcelona (UAB), Bellaterra, Barcelona, Catalonia Spain; 3 Agrupació de Defensa Sanitària de Cabrum i Oví Lleter de Catalunya, Barbens, Catalonia Spain; 4 Departament d’Agricultura, Ramaderia, Pesca i Alimentació, Generalitat de Catalunya, Barcelona, Catalonia Spain

**Keywords:** Tuberculosis, Live attenuated vaccines

## Abstract

Vaccination of goats against tuberculosis (TB) has been promoted as an ancillary tool for controlling the disease in infected livestock herds. A three-year trial to assess the efficacy of BCG vaccine was carried out in five goat herds. At the beginning of the trial (month 0), all animals were tested for TB using thee different diagnostic tests. Animals negative to all tests were vaccinated with BCG and all replacement goat kids were also systematically vaccinated throughout the trial. All animals were tested by Interferon-gamma release assay (IGRA) using vaccine compatible reagents at months 6, 12, 24, and 36. The risk factors for TB infection were also evaluated. At the end of the study, four out of five farms showed variable reductions of the initial prevalence (93.5%, 28.5%, 23.2%, and 14.3% respectively), and an overall incidence reduction of 50% was observed in BCG vaccinated goats, although adult vaccinated goats showed higher incidences than vaccinated goat kids. The unvaccinated positive animals remaining in herds and adult BCG vaccinated goats significantly enhanced the risk of infection in vaccinated animals. A systematic vaccination of goats with BCG, together with the removal of positive unvaccinated animals, may contribute to reducing the TB prevalence in goat herds.

## Introduction

Caprine tuberculosis (TB) is an infectious disease caused by different members of the *Mycobacterium* *tuberculosis* complex (MTBC), mainly by *Mycobacterium* *caprae* and *Mycobacterium* *bovis*^1–3^. Infected goats participate in the epidemiology of animal TB in multi-host settings^4–8^ and pose a risk of zoonotic TB^9^.

In addition, goat TB in endemic herds implies large economic losses due to the reduction of milk production in TB infected goats^10^, and to trade restrictions and depopulation of positive herds^11,12^. In underdeveloped countries or high TB prevalent settings (such as Spain), eradication programs based only on test and slaughter are not always the best cost-effective strategy. In this regard, vaccination of goats against TB has been promoted as an ancillary tool for reducing TB prevalence^13^.

The *M.* *bovis* bacillus Calmette-Guérin (BCG), the only TB licensed vaccine in humans, and badgers in the United Kingdom, has been demonstrated to be safe and relatively inexpensive to produce^14^. The efficacy of BCG against TB has been evaluated in some livestock and wildlife species^15^, yielding variable results^15^. A few field trials have been conducted in cattle^16–20^, goats^21^, and wildlife^22,23^. Although protection was incomplete, encouraging results were observed in reducing the extent of lesions and the transmission of TB.

One of the major challenges for BCG vaccination in livestock is its interference with TB current diagnostic tests, namely the single intradermal test (SIT) or the single intradermal cervical comparative tuberculin test (SICCT) and the Interferon-gamma (IFN-γ) release assay (IGRA). Antigens to differentiate infected from vaccinated animals (DIVA) have been developed in the two last decades to overcome these interferences^24^ and were evaluated in goats in both infection and vaccination settings^25–27^.

Finally, identifying factors that increase or reduce the persistence of TB in infected herds become crucial for eradicating and controlling strategies. Herd size, housing type, or feeding practices have been identified as risk factors for TB persistence in cattle herds^28,29^. Even though previous experimental studies demonstrated that BCG vaccination of goats confers protection against TB^30–32^, the factors influencing TB persistence should be disclosed to assess the efficacy of vaccination strategies at a herd level.

The present study aimed to assess the efficacy of BCG vaccination during three years in five goat farms at a herd level and among vaccination batches, and the identification of the risk factors that may influence the efficacy of a vaccination program for the control of TB in goats.

## Results

### Vaccine efficacy at a herd level

**Figure 1:**
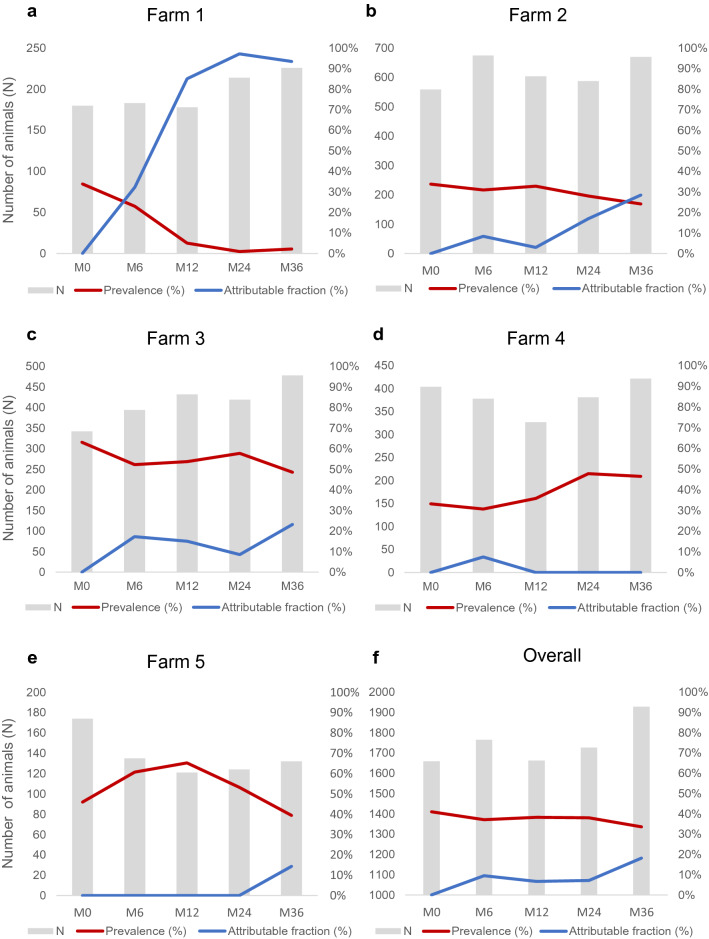
Evolution of prevalence throughout the BCG vaccination trial. Evolution of census (grey bars), prevalence (red lines) and percentage of prevalence reduction (blue lines) in farms 1 (**a**), 2 (**b**), 3 (**c**), 4 (**d**) and 5 (**e**), and overall reduction of five farms (**f**). The prevalence was measured as the percentage (%) of TB positive animals = number of TB positive goats / total census. The reduction of prevalence was measured as attributable fraction (AF) = (% of TB positive at M0—% of TB positive at M6 or M12 or M24 or M36) / % of TB positive at M0.

The evolution of TB prevalence and the prevalence reduction estimated on the basis of the attributable fraction (AF) were variable among farms throughout the vaccine trial (Fig. 1). At the end of the trial (month 36), a favorable outcome was observed in four out of five farms. Overall AF (from month 0 to month 36) of 93.5%, 28.5% and 23.2% were obtained in farms 1, 2, and 3, respectively (Fig. 1a–c). In farm 5, there was a dramatic decrease in the census in the six first months of the trial (from 174 to 135 animals), and the prevalence raised from 46.0% at month  (M) 0 to 60.7% at M6. The overall AF in farm 5 was 14,32% (Fig. 1e). In farm 4, the percentage of positive animals remained stable until M12 (~ 33%) but increased to 46.5% from M12 to M36. Thus, no prevalence reduction was obtained in farm 4 (Fig. 1d). The overall results of the five farms are represented in Fig. 1f. Altogether, the overall initial prevalence (M0 = 41%) started to drop at M6 (37.1%), remained stable at M12 (38.3%) and M24 (38.1%), and continued to drop at M36 (33.6%). The overall AF between M0 and M36 in the five farms was 18.1%.

**Figure 2:**
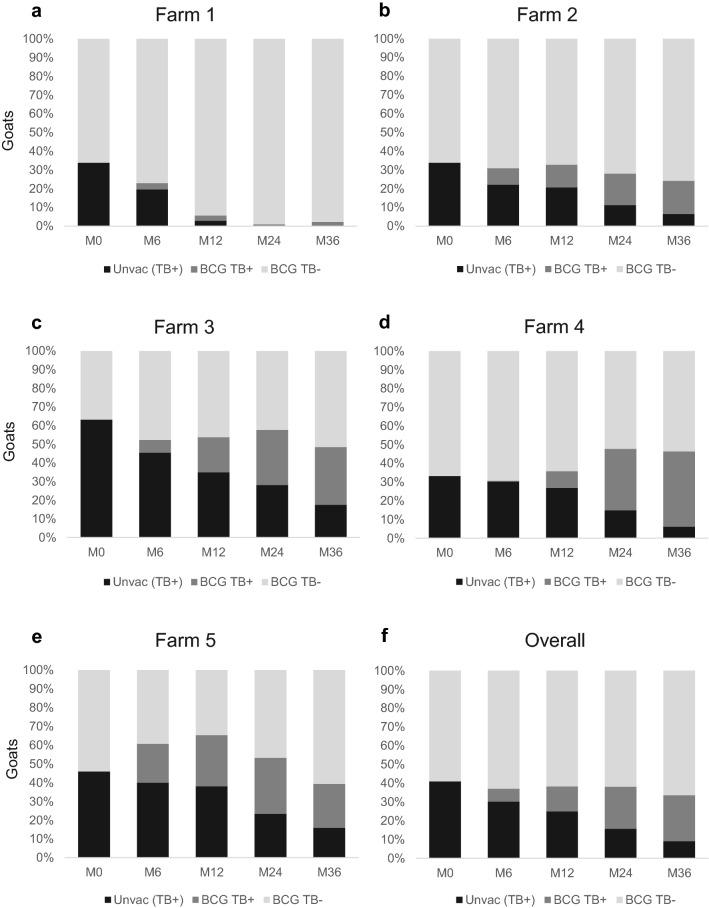
Proportion of TB positive and BCG vaccinated goats from farms 1 (**a**), 2 (**b**), 3 (**c**), 4 (**d**), 5 (**e**) and all farms (**f**) throughout the trial. Evolution of population of vaccinated goats TB negative (light grey), vaccinated TB positive goats (dark grey) and unvaccinated animals (black) during the 36 months (M) of trial.

Farms 1, 2, 3, and 5 showed a significant overall reduction of TB positive animals (*P* < 0.001, chi-square test), passing from 546 TB positives out of 1255 goats at M0 (i.e. 43.5% of TB positive animals) to 451 out of 1506 at M36 (303 TB positive goats vaccinated and 148 TB positive unvaccinated goats), with an overall percentage of prevalence reduction (AF) of 31% at the end of the study. The overall prevalence (% of TB positive animals) and AF of these four farms that showed a favourable vaccination outcome are summarized in Table 1. The overall proportion of TB-infected animals significantly decreased at each sampling time point while the AF increased. The evolution of the goat census in terms of TB status in farms is shown in Fig. 2. In farms with a positive outcome, at M36, the unvaccinated animals that remained in the herds represented 32.8% of the total of TB positive animals. The vast majority of goats (90.2%) from these farms were vaccinated, and 77.7% of them remained TB negative.

**Table 1:** Overall evolution of TB status of farms 1, 2, 3 and 5 throughout 36 months of vaccination trial.

	Month 0	Month 6	Month 12	Month 24	Month 36
Total Census	1255	1387	1335	1345	1506
% TB + animals	44% (33–63)	39% (23–61)*	39% (5–65)*	35% (1–57)**	30% (2–48)**
Attributable fraction^a^	–	11% (0–32)	11% (0–85)	19% (0–97)	31% (14–94)

^a^Attributable Fraction: Percentage of prevalence reduction between prevalence at M0 and the prevalence at subsequent samplings. Ranges are shown in parenthesis. * *P* < 0,05. ** *P* < 0,001. Chi square test. Significant differences respect to the initial proportion of positive animals at M0.

The incidence (Ia) of TB in BCG vaccinated goats from farms was also calculated (Table 2). The overall data from all five farms did not show reductions in the incidence between samplings. In farms with a favourable outcome (farms 1, 2, 3, and 5), in general, there was a progressive reduction of incidence between sampling time points, until the end of the trial, and a 50% of overall incidence reduction was observed from M6 to M36.

**Table 2:** Evolution of TB incidence risk (IR) in BCG vaccinated goats from farms throughout the study.

Farm	M0-M6			M6-M12			M12-M24			M24-M36		
	TB +	Total^a^	IR (%)	TB +	Total	IR (%)	TB +	Total	IR (%)	TB +	Total	IR (%)
1	6	109	6	5	131	4	2	106	2	4	120	3
2	55	360	15	21	454	5	42	369	11	49	576	9
3	23	121	19	53	183	29	32	116	28	27	247	11
4	2	255	1	24	244	10	73	188	39	87	330	26
5	28	90	31	8	53	15	8	38	21	6	88	7

^a^Total of susceptible animals was calculated by the addition of the new vaccinated goat kids minus the half of the number of vaccinated animals that died or were slaughtered between sampling time points (we assumed that they were present a half of the period).

### Efficacy of BCG in the different vaccinated batches

At the end of the trial, in farms 1, 2, 3, and 5, adult goats vaccinated at the beginning of the study showed a higher proportion of TB positive animals compared to the goat kids vaccinated at 2–3 months of age (Table 3).

**Table 3:** TB prevalence at month 36 of trial by age of vaccination of goats from farms 1, 2, 3 and 5.

	TB +	TB-	Total	% TB + at M36
Adult^a^	142	179	321	44%
Goat kids^b^	161	876	1037	16%

^a^Adult goats vaccinated with BCG at M0. ^b^Goat kids vaccinated at 2–3 months of age throughout the trial.

**Figure 3:**
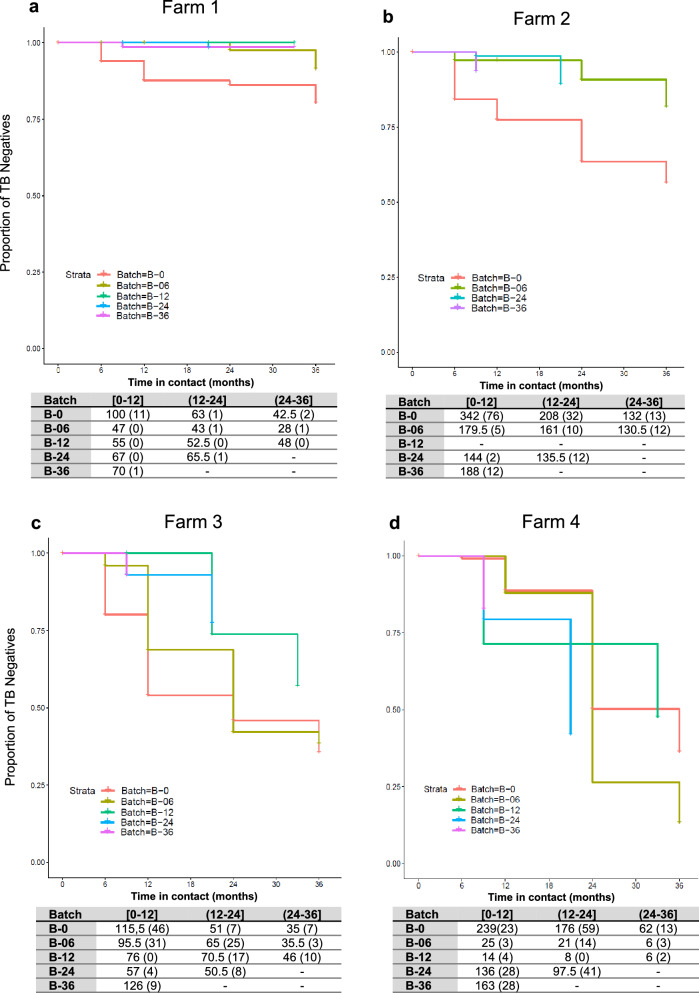

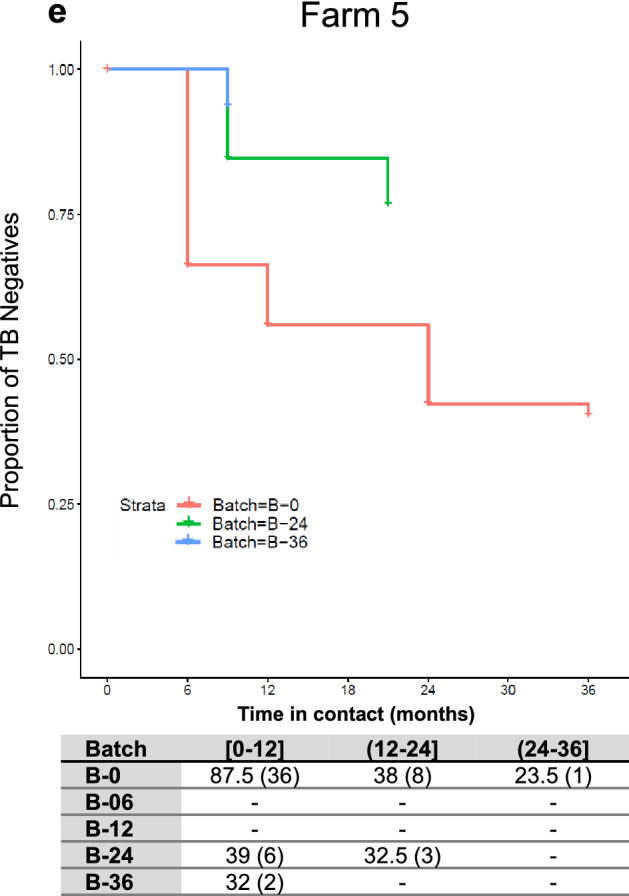
TB Incidence in BCG vaccinated batches from farms 1 (**a**), 2 (**b**), 3 (**c**), 4 (**d**) and 5 (**e**) throughout the trial. Proportion of TB negative animals (y axis) throughout the time in contact (x axis) with the TB positive herd in months. The table represents the incidence of TB in each batch (B) depending on the time in contact, between 0 and 12 months ([0–12]), more than 12–24 months ((12–24]) and more than 24–36 months ((24–36]). The animals at risk, which were the TB negative animals between sampling time points (some animals were slaughtered or died between periods. Those animals were considered present during the half of the period and its number was divided by two). In parenthesis are represented the number of TB new positive goats during a given period.

The evolution of TB status throughout the trial in BCG vaccinated batches is shown in Fig. 3. Among farms, adult goats vaccinated at M0 (Batch B-0) showed the highest TB incidence during the first year in contact with infected animals of the herd regardless of the farm (Fig. 3, B-0 showed the highest decrease in the proportion of TB negative animals during this period). In farms 1, 2, and 5, all batches of vaccinated goat kids (B-06, B-12, B-24, and B-36) showed a low risk of infection irrespectively of the time of contact, with incidences between periods ranging from 0% to 3.5%, 0% to 10% and 6.25% to 9.23%%, for farms 1, 2 and 5, respectively (Fig. 3 a, b and e). In farm 3 (Fig. 3c), the proportion of TB negative adult vaccinated goats at M0 (Batch B-0), and goat kids vaccinated at M0 (Batch B-06) decreased dramatically after the first year of contact, with only 54% and 69% of goats remaining TB negative, respectively. After three years of contact, only 35% and 38% of individuals from batches B-0 and B-06, respectivley, remained TB negative. In addition, in batch B-12 (goat kids vaccinated during the first year of the trial), the proportion of TB negatives dropped to 57% after three years of contact. In contrast, the proportion of TB negative animals in batches B-24 and B-36 (goat kids vaccinated in the second and third year of the trial), that were in the in contact for two and one years of study, respectively, were 77.5% and 93%, respectively. In farm 4 (Fig. 3d), after three years of contact, the proportion of TB negatives within adult vaccinated goats (B-0) decreased to reach a 36% and in animals vaccinated when goat kids, B-06, B-12, decreased to 13% and 47%, respectively. In batches B-24 and B-36, after two and one years of exposure, the proportion of TB negatives was 42% and 83%, respectively.

The TB incidence risk was evaluated using the multilevel model with the lowest AIC. Results of the paired comparisons between batches of the four farms during the first year in contact with the TB positive herd are described in Table 4. The risk of TB in batch B-0 (adult goats vaccinated at M0) during the first year of contact was significantly higher compared to three out of the four batches of vaccinated goat kids (B-06, B-24 and B-36). No differences were observed among batches vaccinated when goat kids, neither among batches after two or three years of contact with TB positive herds regardless of the age of vaccination.

**Table 4:** Risk of TB infection per batch during the first year in contact with the TB positive herd.

Comparisons between batches	Risk Estimate^1^
B-0 versus B-06	0.927 (± 0.186)^a^
B-0 versus B-12	19.78 (± 323.87)
B-0 versus B-24	1.89 (± 0.299)^b^
B-0 versus B-36	1.61 (± 0.219)^b^

^1^Risk estimate coefficient of TB infection (± SE) between batches in farms 1, 2, 3 and 5, determined by a generalized mixed-effect model with post hoc Tukey comparisons between batches. ^a^ *P* = 0.0001, ^b^*P* < 0.0001.

### Factors influencing the incidence of TB in vaccinated animals

The generalized mixed-effect model of each risk factor showed that the qualitative variable of ventilation facilities (high or low), and four quantitative variables (the initial prevalence, the number of positive unvaccinated animals remaining in herds, the number of adult BCG vaccinated TB positive goats at the end of the study and, the number of BCG vaccinated TB positive goat kids at the end of the study) potentially influenced the incidence in vaccinated animals. Thus, these six variables were included in the multilevel model. The initial census and management (intensive or extensive) did not show any relevant effect (*P* > 0.3) at the individual analysis and thus, were discarded for the multilevel model. Based on the multilevel model with the lowest AIC, the number of positive unvaccinated animals remaining in herds and the number of positive adult vaccinated goats (B-0) were significant risk factors (*P* < 0.05) for TB infection in BCG vaccinated animals. The initial herd prevalence was still not a significant TB risk factor (*P* < 0.1). The other parameters included in the model were not detected as significant risk factors for TB infection in BCG vaccinated goat herds.

## Discussion

In this field study, the efficacy of long-term vaccination with BCG of adult and replacement goat kids was evaluated. The vaccine efficacy was determined as the reduction of prevalence, the AF, and the reduction of incidence of each herd during three years, by using IGRA responses against DIVA antigens. The results of vaccine efficacy were heterogeneous among farms, similar to that observed in previous human studies^33^, varying from substantial to absence of protection. Taking into account the data from all farms, the moderate vaccine efficacy observed in the present study was similar to that observed in a systematic review of BCG vaccination trials in children^34^. Protection of goat kids against TB with BCG has been previously reported in a preliminary study conducted in field conditions, as a reduction in the number of animals with TB lesions in the vaccinated group compared to the unvaccinated one (35% and 77%, respectively), after 18 months of exposure to natural infection in the herd^21^. In the light of these results, the present study was aimed to assess the long-term efficacy of a blanket vaccination program, in a real field situation with productive goats, in which all susceptible animals (TB negative adults and replacement goat kids) were vaccinated. Four out of five goat farms (farm1, 2, 3, and 5) showed a favourable outcome in terms of vaccine efficacy (AF, prevalence and incidence reductions), with a majority of vaccinated animals remaining negative. Other field trials to assess BCG efficacy in cattle showed an even higher proportion of protected animals, although it was determined using different parameters. Thus, protections of 77.9% and 67.4%, measured as TB lesions and bacterial load reduction, were observed in subcutaneously (BCG 3 × 10^5^ CFU) and orally (BCG 1 × 10^8^ CFU) vaccinated cattle, respectively^17,18^.

Comparisons of BCG efficacy between field trials should be addressed with caution since the studies were conducted under different conditions, animals were exposed to different TB infection rates, and efficacy was measured using different parameters. Here, the initial TB prevalence of farms ranged between 33–63%, whereas in cattle studies, the herds had a proportion of TB positive reactors under 6%. These differences in herd TB prevalence may explain the higher BCG efficacy rates described in cattle than in the present study in goats. Another explanatory factor is the apparent higher intra-herd transmission of TB within the goat herds of the present study. Most of the herds (four out of five) had highly intensive management which implies that animals are grouped, often in high densities, and usually remaining in buildings with or without access to the outdoors, in contrast to extensive management where densities are low and animals usually have permanent access to outdoors for grazing. Thus, this intensive management in farms may have entailed closer contact among animals, with the exception of farm 1, in which the vaccine showed the highest protection rate and the management was extensive.

Even though BCG vaccination does not provide full protection against TB, it reduces the transmission of MTBC^15^. Previous field and experimental studies in cattle and goats showed that BCG decreased either mycobacterial shedding^16^ or TB pathology^20,21,31^. Different practices may affect the evolution of incidence and prevalence among goat farms. All farmers discarded (more or less intensively) the old unvaccinated TB positive animals (results of tests were made available) for the slaughterhouse, which is one of the most common control measures. In this sense, Ameni and collaborators suggested that gradual removal of positive animals and their replacement by BCG vaccinated cattle, can be a valuable approach for TB control when intensive test and cull of positive animals is not affordable^19^. Accordingly, the transmission of *M.* *caprae* in the present field trial, measured as TB incidence in vaccinated animals, was progressively reduced in four out five farms, attaining an overall reduction of 50% (Table 2).

Intriguingly, in farm 5, even with a positive outcome at the end of the trial, the initial prevalence increased six months after vaccination. In this case, a reduction of the census and a raise of incidence among vaccinated goats were observed. This farm had the lowest census among farms from the study and these fluctuations in the number of animals had a high impact on the prevalence outcome. Some deaths might be explained by productive reasons (8 goats from B-0 were discarded) and by the cold weather, which can worsen the symptoms of respiratory diseases^35^. These weather conditions might force the farmer to maintain animals enclosed. In this particular case, the buildings had poor airflow, and the proximity between animals probably promoted TB transmission and other respiratory pathogens. To our disappointment, the vaccine failed to confer protection on farm 4. A single factor cannot explain this outcome. However, the results lead us to speculate that the most likely cause of vaccine failure might be that some batches of goat kids could be infected before vaccination. Those animals were vaccinated after two months of age, most of them were in contact with adult goats after birth, and were potentially fed with unpasteurized colostrum. Nevertheless, overall findings suggest that the BCG vaccine is effective in reducing TB prevalence in goat farms. However, its effects are highly influenced by intrinsic factors of the farm (i.e., management practices, facilities, etc.).

The direct effect of vaccination among batches was also evaluated. For that purpose, incidence and time to IFN-γ conversion (DIVA test) of each animal were measured. TB incidence in adult vaccinated goats (batch B-0) was higher compared to vaccinated goat kids (batches B-06, B-24, and B36), mainly during the first year in contact with the TB positive herd. Similarly, vaccination of yearling buffaloes with BCG failed to protect them against *M.* *bovis* challenge^36^. The lack of efficacy of BCG in adult buffaloes was probably due to contact with environmental mycobacteria (EM). Indeed, adult goats had more chances to be sensitized by the exposure to EM prior to vaccination than goat kids. Contact with EM before BCG vaccination was associated with reduced vaccine efficacy in cattle^37^ and humans^38^. In addition, goats from five farms were usually infected by helminths. Pre-existing infections with helminths may reduce the efficacy of vaccines against TB^39,40^. Indeed, BCG vaccinated cattle co-infected with *Fasciola* *hepatica* and *M.* *bovis* showed lower IFN-γ responses against PPD-B and reduced the number of reactors to SICCT^41^. Helminthic infections usually drift the immune response towards a type T-helper 2 response and downregulate the T-helper 1^42^. The latter immune response is necessary for the production of IFN-γ and effective responses against *M.* *bovis*^43^. These responses are triggered after BCG vaccination, but it seems that pre-existing infections with helminths do not lead to the development of those responses^43^. Another relevant factor that cannot be ruled out is that some animals vaccinated at the beginning of the study (B-0) after showing a negative result to the IFN-γ DIVA test could be false negatives. In fact, chronic TB infections inducing anergy have been observed early in life in goats^44^ and in young calves^45^ probably due to the exhaustion of antigen-specific cell-mediated immune responses against the mycobacteria after a long period of infection^46^.

The multilevel model detected a higher risk of TB infection in adult vaccinated goats (B-0) during the first year in contact with the TB positive herd. The risk was significantly higher compared to B-6, B-24, and B-36. Differences between B-0 and B12 were not significant, probably because only two farms had a batch B-12, and the number of goats in this batch was low (see Fig. 3a and 3c). These findings also reinforce the hypothesis that vaccination of adult goats could be less effective. In addition, the incidence and the risk of TB infection in replacement goats detected by the multilevel model were similar regardless of the time of contact and the batch of vaccinated goat kids. Nonetheless, after one year of contact, the risk of TB infection was similar among batches irrespectively of the age of vaccination. Thus, the protective immunity of BCG vaccination decayed after one year. In the same way, in BCG vaccinated cattle, no significant protection by the reduction in gross pathology was observed after challenge with *M.* *bovis* after 24 months of BCG vaccination^47,48^. However, significant protection was still detected when the challenge was carried out at 12 months post-vaccination^47^. Here, despite a decrease in BCG vaccine efficacy after one year of vaccination regardless of the batch, some degree of individual protection may remain, and systematic vaccination of replacement goat kids, progressively reduced the TB prevalence, as observed in oral BCG vaccinated badgers^23^. It suggests that high vaccination coverage may increase the protection against TB infection within the herd and improve the vaccine efficacy. Even so, other strategies to boost the protective immunity against TB, such as BCG revaccination, that showed improved protection than the single-dose regime in cattle^48^, might be further explored in large scale field trials.

On the other hand, as mentioned before, other control measures and intrinsic factors from farms may contribute to the control of TB and may favour the success or failure of the vaccine program. In this context, the multilevel model was also used to evaluate different factors influencing the TB incidence among farms. The TB positive unvaccinated animals remaining in the herds and the adult vaccinated goats were identified as the two factors that increased the risk of TB cases in herds. As expected, initial TB positive animals were a source of infection for vaccinated animals. Indeed, some adult animals, probably chronically infected, may act as super shedders^49^. Regarding the risk of adult vaccinated goats, as previously observed in humans, vaccination of adults was not protective^33^. Moreover, as discussed above, it is possible that some vaccinated animals were anergic before BCG vaccination, which is a phenomenon that can appear relatively early in goats (at 2–3 years of age)^44^. Thus, despite vaccination, in our conditions, adult vaccinated goats may act as the unvaccinated goats, contributing to the spread of the TB infection in herds. On the other hand, the initial prevalence was not yet detected as a significant risk factor for TB infection in the herds, however, a high initial prevalence may increase the pressure of infection, hampering the positive effects of vaccination. Interestingly, BCG vaccinated goat kids that converted positively to IGRA did not appear to represent a risk for TB infection for other vaccinated animals. Probably these goats were less likely to infect other animals because they were partially protected and did not excrete or had reduced the mycobacterial shedding^16^. Also, previous experimental infections with *M.* *caprae* showed slower progression of the TB infection, and a lower degree of lung lesions in BCG vaccinated goats compared to unvaccinated controls^30–32,50^. This reduction of pathology does not favour the mycobacterial excretion by coughing^51^. Thus, both the progressive slaughter of TB positive unvaccinated animals and the systematic BCG vaccination of replacement goat kids may contribute to the reduction of TB incidence and TB prevalence in goat farms. Furthermore, previous studies have identified a high census of cattle^28,52^ and intensive management^53^ as risk factors for TB disease. However, in our conditions, these factors did not show a significant impact on the risk of TB infection. It is important to remark that risk assessments should be taken with caution because sample size, the measures used, the variables analysed, and the size of herds may differ among studies^28^.

Finally, the SICCT and the IGRA with tuberculins and DIVA reagents were used as indicators of TB infection in order to maximize the detection of infected animals prior to vaccination. However, as BCG vaccination causes interferences with the SICCT and the IGRA standard, at least after one year of vaccination^47^, the only available diagnostic tool for the diagnosis of TB in vaccinated goats was the IGRA using DIVA reagents. The main caveat of this DIVA test in goats is that, the observed sensitivity in previous studies in TB infected goat herds was around 70%^25,27^, despite being highly specific (100%)^27^. On the other hand, as responses developed against BCG vaccine contribute to the containment of the infection^30^, it is possible that some vaccinated-infected animals do not develop a detectable response by the IGRA DIVA. Thus, up to 30% of false negatives among vaccinated animals had to be assumed in the present study and this could attenuate the intervallic incidence risk. However, the same criterion for positivity was used in all time-points, enabling to assess the vaccine efficacy by the evolution of TB prevalence and AF.

In conclusion, our results showed that systematic BCG vaccination of replacement goat kids could contribute to reducing the transmission of *M.* *caprae*, steady reducing the overall TB prevalence within the herds, even when individual vaccine efficacy decreased one year after vaccination. Moreover, removing positive unvaccinated goats from the herds reduced the risk of new TB cases, favouring the success of the vaccination program. Therefore, besides vaccination, additional measures, such as test and cull of positive animals, are strongly recommended. The present study provides relevant data to evaluate the cost–benefit of a long-term vaccination strategy to the control of caprine TB.

## Material and methods

### Study design and animals

The present research was carried out in the framework of preliminary studies to establish an action plan for the control and eradication of caprine TB of the Department of Agriculture, Livestock, Fisheries, and Food of the Catalan Government (DARP). It was a field trial including five goat farms from Catalonia (Spain) with confirmed TB infection by culture (M. caprae) from tuberculous lesions in necropsied goats. Farms were selected due to the high proportion of positive animals detected in an initial screening carried out by the single-intradermal cervical comparative skin test (SICCT) (above 30% of positivity). The M. caprae strains (www.Mbovis.org) from each farm are detailed in Table **5**. Farms had different sizes, production, and management characteristics.

**Table 5:** Characteristics of goat farms.

Farm	Breed	No. of animals	Production	Management	Replacement origin	*M.* *caprae* spoligotype
1	Blanca de Rasquera	180	Meat	Extensive	Own	SB0415
2	Malagueña	557	Milk	Intensive	Own	SB0157
3	Murciana-granadina	458	Milk	Intensive	Mixed	SB0415 SB0157
4	Murciana-granadina/Alpine/mixed	434	Milk	Intensive^1^	Mixed	SB0415
5	Mixed breeds	174	Milk	Extensive	Own	SB0415

^1^Ecological farm (daily grazing for 8 h, no use of antibiotics).

The trial was conducted for 36 months. At the initial test sampling (Month 0), all adult and young (> 6 months) goats were tested with SICCT and IGRA, using M. bovis and M. avium tuberculins (CZV vaccines, Porriño, Spain) and a reagent for differentiating infected from vaccinated (DIVA) based on ESAT-6 and CFP-10 MTBC antigens (Lionex, Braunschweig, Germany). Animals positive at least to one test were classified as positive, and animals negative to all tests were vaccinated subcutaneously with BCG Danish 1331 strain (ATCC, Ref. 35733) behind the axillar region. All replacement goat kids added to the herd during the trial were systematically vaccinated at 2–3 months of age, and batches (B) were named depending on the month of first entry on sampling routine (B-06, B-12, B-24, and B36, respectively). Replacement animals were managed in the herd following the productive routine of each farm. All goats with more than six months of age were tested at months (M) 6, 12, 24, and 36 after the initial vaccination (at Month 0) by the IGRA with tuberculins and DIVA reagents. BCG vaccinated animals were considered as positive depending only on the results of the DIVA test.

### Vaccine

The M. bovis BCG Danish 1331 strain (ATCC, Ref. 35,733) was prepared as described previously^30^. Briefly, an aliquot of BCG was subcultured in the Middelbrook 7H9 medium (BD diagnostics, sparks MD USA) and incubated for 28 days at 37 °C. Then, an aliquot of the growth culture was tenfold diluted on phosphate-buffered saline (PBS) with 0.05% Tween 80, and plated on 7H11 solid media (BD diagnostics) and incubated for 28 days at 37 °C, then the remaining growth culture was divided in 1 ml aliquots that were stored at -80 °C. After incubation, the bacterial count was performed. For vaccine preparation, a suspension of one aliquot of growth culture was thawed and diluted in PBS to reach approximately10^6^ CFU/ml. An aliquot of 0.5 ml of this suspension was subcutaneously injected in goats, and an aliquot of 70 µl of the suspension was tenfold diluted to verify the titer of the vaccine (2–8 × 10^5^ CFU/ml).

### Single-intradermal cervical comparative skin test (SICCT)

Before intradermal inoculation, the area was shaved, and skinfold thickness (SFT) was measured. The SICCT was performed by intradermal inoculation of 0.1 ml of purified protein derivative from *M.* *bovis* (PPD-B) and *M.* *avium* (PPD-A) at 25,000 IU/ml each one*,* at the left-hand side and the right-hand side of the neck, respectively, by using a Dermojet syringe (Akra Dermojet, Pau, France). The increase of SFT was measured 72 h after inoculation. SICCT was interpreted as recommended by the Spanish bovine TB eradication program^54^. A goat was considered a positive reactor if SFT PPD-B > 2 mm and SFT PPD-B—SFT PPD-A > 1 mm, or if the presence of clinical signs in the PPD-B inoculation site.

### Whole blood interferon-gamma release assay (IGRA)

Blood samples from the jugular vein were collected in heparinized tubes and were processed under 8 h of bleeding, as described previously^26^. Briefly, blood samples were stimulated with PPD-A, PPD-B, and the DIVA reagent (ESAT-6/CFP10 cocktail) at a final concentration of 20 μg/ml each. PBS was used for the unstimulated control. Samples were then incubated overnight at 37 ± 1 °C with 0.5% CO_2._ Plasma supernatants were collected by centrifugation and analysed by ELISA (BOVIGAM, Thermo Fisher Scientific, Waltham, MA, USA) and read at 450 nm using a spectrophotometer (Biotek Power Wave XS). The tuberculin IGRA based test was interpreted as recommended by the manufacturer: an animal was considered positive if PPD-B OD – PBS OD ≥ 0.05 and PPD-B OD > PPD-A OD, and for the DIVA reagent, an animal was considered positive if ESAT-6/CFP-10 OD – PBS OD ≥ 0.05.

### Parameters considered for the analysis

In order to assess the effects of BCG vaccination, different parameters were calculated:

- The prevalence (P), estimated as the percentage of TB cases (new and old) at each “i” sampling time.
- Prevalence reduction between each follow-up period: estimated on the basis of the attributable fraction (AF) between periods as follows:
  $${\text{AF}}\left( {{\text{i}} + 1} \right) = \frac{{{\text{P}}\left( {\text{i}} \right) - {\text{P}}\left( {{\text{i}} + 1} \right) }}{{{\text{P}}\left( {\text{i}} \right)}}$$
- The incidence risk (IR) in BCG vaccinated animals at each “i” sampling time. In order to account for the animals that entered (new) or leaved (lost) the group of vaccinated animals (N) throughout each follow-up period, we followed this approximation:
  $${\text{IR}}\left( {{\text{i}} + 1} \right) = \frac{{{\text{New}}\,{\text{cases}}\,\left( {{\text{i}} + 1} \right) }}{{{\text{N}}\left( {\text{i}} \right) + {\text{Replacement}}\,{\text{goat}}\,{\text{kids}}\,\left( {{\text{i}} + 1} \right) - \frac{1}{2}*{\text{Lost}}\left( {{\text{i}} + 1} \right)}}$$

### Statistical analysis

Differences between the number of TB positive animals at the beginning of the study (Month 0) and the end of the study (Month 36) for each studied farm, were calculated using a chi-square test. A *P*-value < 0.05 was considered as significant.

In addition, differences in TB incidence risk among BCG vaccinated batches was evaluated taking into account the time in contact with infected animals of the herd of each vaccinated goat (i.e., less or more than one year, more than one year to two years, and more than two years to three years of being introduced in the TB positive farm) and the batch of animals. The time in contact was calculated based on the time that each animal remained negative on the farm. The interaction between both variables was included in the analysis. Besides, two qualitative variables (i.e., intensive or extensive management, high or low ventilated facilities) and five quantitative variables (i.e., number of unvaccinated animals in herds at the end of the study, initial prevalence, initial census, vaccinated positive adult and goat kids at the end of the study) were also considered. At a first step, we fit a generalized mixed-effect model with a Poisson error structure (as the number of cases is a count variable) separately for each variable. Variables with significant influence (*P* < 0.05) or with *P* < 0.3, were selected. In a second step, a multilevel model, also with a Poisson error structure, was fit, which included the farm as a random effect to take into account the lack of independence of the observations. Model selection was based on the Akaike information criterion (AIC), starting with a complex model including all variables with significant influence. Then, the complexity of the model was reduced by removing one by one of the variables without significant influence (based on *P*- values > 0.05). The AIC’s of models generated were compared. Finally, the model with the lowest AIC was selected, and Tukey comparisons were performed in order to compare the incidence risk among batches depending on the time of exposure. All the analyses were performed using RStudio Team (2019). RStudio: Integrated Development for R. RStudio, Inc., Boston, MA URL: https://www.rstudio.com/.

### Ethics approval

All procedures were approved by the Research Ethics Commission of the Generalitat de Catalunya (procedure number 8697). All animals were managed by the personnel of the farm following conventional procedures and qualified veterinarians, authorized by the Departament d’Agricultura, Ramaderia, Pesca i Alimentació de la Generalitat de Catalunya (DARP), performed testing and sampling according to European (86/609/CEE) and Spanish (RD 53/2013) legislation.

## Data Availability

The databases generated and analyzed during the current study are available from the corresponding author on a reasonable request.
